# Active Role of ZnO Nanorods in Thermomechanical and Barrier Performance of Poly(vinyl alcohol-*co*-ethylene) Formulations for Flexible Packaging

**DOI:** 10.3390/polym11050922

**Published:** 2019-05-26

**Authors:** Francesca Luzi, Alessandro Di Michele, Luigi Torre, Debora Puglia

**Affiliations:** 1 Civil and Environmental Engineering Department, University of Perugia, UdR INSTM, Strada di Pentima 4, 05100 Terni, Italy; francesca.luzi@unipg.it (F.L.); luigi.torre@unipg.it (L.T.); 2 Physic and Geology Department, University of Perugia, Via Pascoli, 06123 Perugia, Italy; alessandro.dimichele@unipg.it

**Keywords:** zinc oxide nanorods, poly (vinyl alcohol-co-ethylene), weathering

## Abstract

Poly(vinyl alcohol-co-ethylene) (EVOH) films containing zinc oxide nanorods (ZnO Nrods) at 0.1, 0.5, and 1 wt%, were realized by solvent casting. The effect of ZnO Nrods content on morphological, thermal, optical, mechanical, and oxygen permeability properties were analyzed. In addition, moisture content and accelerated-aging test studies were performed, with the intention to determine the influence of zinc oxide nanofillers on the functional characteristics of realized packaging systems. Tensile properties showed increased values for strength and deformation-at-break in EVOH-based formulations reinforced with 0.1 and 0.5 wt% of zinc oxide nanorods. Results from the colorimetric and transparency investigations underlined that the presence of ZnO Nrods in EVOH copolymer did not induce evident alterations. In addition, after the accelerated-aging test, the colorimetric test confirmed the possibility for these materials to be used in the packaging sector. This behavior was induced by the presence of zinc oxide nanofillers that act as a UV block that made them useful as an efficient absorber of UV radiation.

## 1. Introduction

Over the previous two decades, polymer nanocomposite-based formulations, which are particle-filled polymeric systems with at least one dimension of the dispersed fillers in the nanometer range, have received a lot of interest from industrial and academic researchers. The addition of inorganic nanoparticles (usually at low content up to 5 wt%) into a polymer matrix improves several properties of the material with respect to the polymeric phase. The main characteristics are thermal [1,2], heat resistance [3,4,5], electrical [6], mechanical [7], and barrier properties, for nanocomposites with low nanofiller content (approximately 3% and 5%) [8]. These improved characteristics are related to the polymer selected to realize the nanocomposites and also to the morphological aspect of nanofillers (large interfacial area and supramolecular organization), which are important properties to reach the desired macroscopic characteristics.

The packaging sector in recent years has received much attention because it provides some level of protection to products from internal and external unfavorable conditions [9,10].

Among the polymers used in packaging, poly(vinyl alcohol-co-ethylene) (EVOH) can be certainly considered as one of the most suitable polymeric matrixes to realize packages [11,12]. The popularity of this polymer can be related to its specific characteristics. EVOH is a semicrystalline random copolymer with excellent chemical resistance [12], high transparency, and gas barrier properties [13,14], particularly for EVOH polymers with a low content of ethylene (below 38 mol% ethylene) [15]. A negative aspect of EVOH copolymers is their moisture affinity, which influences a significant reduction of their characteristics (mechanical, thermal, and barrier characteristics) at high relative humidity environments [16]. Specifically, in dry conditions the barrier and mechanical properties of EVOH are related to the high inter- and intra-molecular cohesive energy and semi-crystalline morphology [17]. Other than food, EVOH has found wide application also in the electronic packaging sector, as an attractive option for the encapsulation of flexible organic electronic devices [18], in a multilayer approach in combination with PE or PP [19].

Unfortunately, the stability of these devices is still a challenge due to the low resistance of cell components against the presence of oxygen and moisture. However, ongoing research on encapsulation has shown that device lifetime can be considerably prolonged by using appropriate materials both for rigid and flexible coatings [20,21]. Consequently, one option to be considered for maintaining stability of such electronic devices is the encapsulation with polymeric films having high barrier properties, which can prolong device life. In the field of industrially available polymers, EVOH can represent a solution for its easy processing and unpaid barrier properties. On the other hand, other environmental factors, such as UV radiation and moisture, can limit material performance, so some strategies based on a nanotechnological approach have been proposed and implemented to advance the weathering performance of plastics.

It has been extensively proved that oxide particles like titanium dioxide (TiO_2_), zinc oxide (ZnO), cerium oxide, and iron oxide have the ability to protect (by absorbing and scattering) from UV radiation [22]. Such capabilities can be utilized to improve the photo-stability of various polymeric materials [23]. It has been demonstrated that inorganic metal oxides have increased functional and structural properties of polymeric-based matrices [24,25,26]. Among the metal oxides, ZnO is one of the most extensively investigated materials in numerous fields due to its noteworthy photocatalytic, antifungal, and antimicrobial characteristics [10,26]. ZnO nanoparticles, in comparison to other metal oxide nanoparticles, are considered as safe materials for humans, and have been used as food additives, in water purification, and in packaging materials [27,28]. They are considered environmentally friendly for various polymers, providing some interesting properties like intensive ultraviolet absorption and an antibacterial effect. ZnO has been utilized in a number of packaging coatings, especially in the food sector, to increase desirable packaging characteristics, including a thermomechanical stability barrier and, specifically, mechanical strength properties [29,30].

Nano-sized ZnO fillers have also shown biocidal activity and other some positive aspects with respect to other metallic and organic/inorganic nanoparticles, such as their white appearance, low cost, and UV blocking properties. ZnO is colorless with an optical band gap in the UV region that makes it advantageous as an efficient absorber of UV radiation and as a wide band-gap semiconductor [31].

The aim of this research was the development, production, and characterization of EVOH and EVOH_ZnO Nrods (zinc oxide nanorods)-based formulations for packaging applications. In this work, zinc oxide nanorods (0.1, 0.5, and 1 wt%) were utilized as UV block that made them useful as an efficient absorber of UV radiation. EVOH films were prepared by solvent casting techniques.

The formulations were characterized in terms of morphological, optical, thermal, and mechanical properties. Functional characteristics of relevant importance for the packaging sector, such as moisture content and oxygen permeability, were examined. Finally, an accelerated-aging test was performed at different times, and then the chemical and optical properties were analyzed to evaluate how ZnO Nrods as UV radiation absorbers could alter the good performance of EVOH film towards UV weathering and photoaging.

## 2. Materials and Methods

### 2.1. Materials

Poly(vinyl alcohol-co-ethylene) with 32 mol% ethylene content (EVOH 32) (density: 1.19 g/mL at 25 °C, melt index: 3.8 g/10 min (210 °C)), 1-Propanol reagent, ≥99.5%, magnesium nitrate (Mg(NO_3_)_2_), zinc acetate, and hexamethylenetetramine were provided from Sigma-Aldrich^®^ (Milan, Italy).

### 2.2. Zinc Oxide Nanorods Synthesis and Characterization

ZnO nanorods were synthesized by using zinc acetate and hexamethylenetetramine both 0.01 M, the materials were sonicated with high power horn-type ultrasound generator (Ultrasonic processors VC750 Sonics and Materials, 20 KHz with a diameter tip of 13 mm) at 90 °C for 1 h at 50% of amplitude. After synthesis the white precipitate was centrifuged, washed with deionized (DI) water and then dried at room temperature. The morphology and the size of the ZnO nanorod was observed by field emission scanning electron microscopy (FESEM) (LEO 1525 ZEISS, Oberkochen, Germany).

### 2.3. Production of EVOH-Based Systems

EVOH and EVOH_Nrods-based films combined with zinc oxide nanorods (Nrods) at 0.1, 0.5, and 1 wt% (EVOH_0.1ZnO Nrods, EVOH_0.5ZnO Nrods and EVOH_1ZnO Nrods) were prepared by solvent casting, method reported in literature [32]. First of all, EVOH was dissolved in 1-propanol/water (70:30 *w*/*w*) in the ratio 8:92 (*w*/*w*) under magnetic stirring at 100 °C for 2 h.

EVOH_Nrods formulations were obtained by mixing EVOH solution (cooled down to approximately 60 °C) with a specific amount of zinc oxide nanorods under magnetic stirring at RT for 1 h. Finally, EVOH solutions were cast in a *Teflon^®^* Petri dish and dried in an oven at 60 °C for 3 h. The films (of 140 mm diameter and 50–70 μm thick) were stored and equilibrated for 2 days in a desiccator by using silica salt, after the processing and before the characterizations [33].

### 2.4. Characterization of EVOH_Nrods Formulations

#### 2.4.1. Morphological, Spectroscopic, and Color Analysis

The microstructure of EVOH and EVOH_Nrods formulations fractured surfaces was investigated by FESEM (LEO 1525 ZEISS) after chrome sputtering and by using an accelerating voltage of 15 kV.

The transparency of different EVOH films was investigated by UV-Vis spectroscopy in the range 250–900 nm by using a Perkin Elmer Lambda 35.

Fourier infrared (FT-IR) spectra of EVOH and EVOH_ZnO Nrods systems were also analyzed using a Jasco FT-IR 615 spectrometer (Jasco Inc, Easton, MD, USA) in the 400–4000 cm^−1^ range in attenuated total reflection (ATR) mode.

Color parameters of EVOH and EVOH_Nrods formulations from CIELAB color space were evaluated by means of a spectrophotometer (CM-2300d Konica Minolta, Japan). SCI 10/D65 method was considered. The diff erent produced films were placed on a white standard substrate and L*, a*, and b* parameters were measured. Three measurements were obtained at arbitrary positions on each of the films. The total color difference ΔE* between EVOH-based systems was evaluated by the following equation:

(1) $$\Delta E*=\sqrt{{\left(\Delta L*\right)}^{2}+{\left(\Delta a*\right)}^{2}+{\left(\Delta b*\right)}^{2}}$$

Gloss value was also determined by using the SCI 10/D65.

#### 2.4.2. Thermal Characterization

Thermal characterization of EVOH systems was carried out by thermogravimetric analysis (TGA) and differential scanning calorimetry (DSC). Thermogravimetric measurements were done by using a Seiko Exstar 6300 instrument, under an inert nitrogen flow (150 mL/min) by heating the samples from 30 to 600 °C at a rate of 10 °C min^−1^.

Differential scanning calorimetric measurements were done on a TA Instruments DSC Q200 (TA Instruments Inc., New Castle, DE, USA) under an inert nitrogen flow (50 mL/min) by using one cooling and two heating scans, between −25 and 210 °C at 10 °C min^−1^. The glass transition temperature (*T_g_*) was measured both in the cooling and the second heating scan, while crystallization temperature and enthalpy (*T_c_* and Δ*H_c_*) were detected in the cooling scan. Melting temperature and enthalpy (*T_m_* and Δ*H_m_*) were also measured and registered for the second heating scan curves.

The crystallinity degree was evaluated according to Equation (2):

(2) $$\chi =\frac{1}{\left(1-m_{f}\right)}\left[\frac{\Delta H}{\Delta H_{0}}\right]*100$$

where ΔH is the enthalpy for melting or crystallization; ΔH_0_ is melting enthalpy for a 100% crystalline EVOH sample and (1 − *m_f_*) is the weight fraction of EVOH in the sample.

The melting enthalpy at 100% of EVOH was calculated according to Equation (3) [34]:

(3) $$\Delta H_{0}=\alpha \Delta H_{0}^{PVA}+\beta \Delta H_{0}^{PE}$$

where Δ*H_0_*^PVA^ is enthalpy of melting for a 100% crystalline of poly(vinyl alcohol) (PVA) taken as 161.1 J g^−1^ [35], while Δ*H_0_*^PE^ is enthalpy of melting for a 100% crystalline of polyethylene (PE) taken as 290.0 J g^−1^ [34] α and β are the weight fraction of vinyl alcohol (α = 0.68) and ethylene (β = 0.32) in EVOH. Δ*H_0_* melting enthalpy for a 100% crystalline EVOH is taken as 202.4 J g^−1^.

#### 2.4.3. Mechanical Characterization

The mechanical properties of EVOH and EVOH Nrods-based films were estimated by tensile tests (UNI ISO 527 standard), at room temperature, of rectangular samples (50 × 10 mm) (five repetitions) with a load cell of 500 N, an initial gauge length of 25 mm and a crosshead speed of 5 mm min^−1^. Young’s modulus (*E_Young_*), tensile strength (*σ_B_*), and elongation at break (*ε_B_*) values were calculated from the stress–strain curves.

#### 2.4.4. Moisture Content and Oxygen Barrier Properties

The moisture content (MC) of EVOH-based films was evaluated by considering 53% RH and 25 °C. Initially, samples were dried in a vacuum oven at 40 °C for 72 h, subsequently pre-dried materials were placed in desiccators containing Mg(NO_3_)_2_ salts until constant weight. Two different times (1 and 5 weeks) and three samples for each formulation were analyzed. MC was estimated according to the Equation (4):

(4) $$MC(\%)=\frac{W_{Final}-W_{Initial}}{W_{Final}}*100$$

where *W*_Final_ is the weight of sample after 1 or 5 weeks at 53% RH and 25 °C and *W*_Initial_ is the initial weight of different samples dried in a vacuum oven at 40 °C for 72 h.

Oxygen transmission rate (OTR) tests were performed in an Oxygen Permeation Analyzer (Model 8000, Systech Instruments, Metrotec S.A, Spain). Films having homogeneous thickness values were tested at 23 ± 1 °C, under an oxygen flow (≥99.9% purity) purged at 2.5 bar (tests were performed in triplicate). The oxygen volumetric flow rate per unit area of the film and time unit (OTR, cm^3^ m^−2^ day^−1^) was constantly monitored until the steady state was obtained. Being the permeability coefficient dependent on the film thickness and proportional to OTR*e (e = thickness, mm), oxygen barrier properties of all the studied films values were evaluated by comparing OTR*e values.

#### 2.4.5. Weathering Test

The polymeric-based systems were exposed to UVA radiation (340 nm, energy of 0.77 W/m^2^) at 60 °C in accordance with the literature [8,21] for 10 days by using Q-Lab (QUV/Spray).

The effect of the accelerated weathering test on the different systems was evaluated in terms of color variation and FT-IR analysis after 5 and 10 days of exposition. More in detail, three samples per type were exposed to the test. The first series of films was extracted out of the machine after 5 days and the second series was extracted after 10 days.

## 3. Results

### 3.1. Nrods Characterization

**Figure 1:**
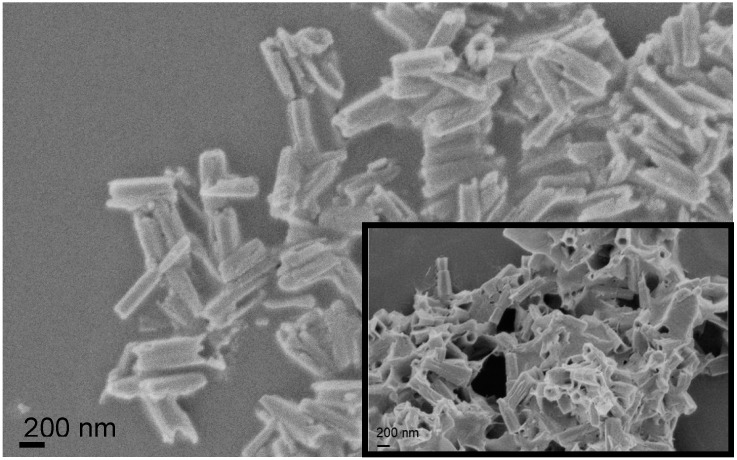
Field emission scanning electron microscopy (FESEM) image of zinc oxide nanorods (ZnO Nrods).

The morphological structure of ZnO Nrods was evaluated by FESEM. FESEM images of the zinc oxide nanorods grown products in Figure 1 reveal that the structures are hexagonal-shaped, in accordance with the literature [36,37]. The surface of each nanorod is clean and smooth [38]. In addition, ZnO Nrods showed a hollow structure (see Figure 1 (insert)). As previously observed in literature, the typical diameters of the grown nanorods are (120 ± 20) nm [36], while the length was around (400–500) nm.

### 3.2. Morphological, Spectroscopic, and Color Analysis of EVOH and EVOH_Nrods Films

Morphological, transparency, and color are the most important characteristics of the polymeric films, especially in packaging sector. The final characteristics, in fact, can influence the commercial success and consumer acceptance.

**Figure 2:**
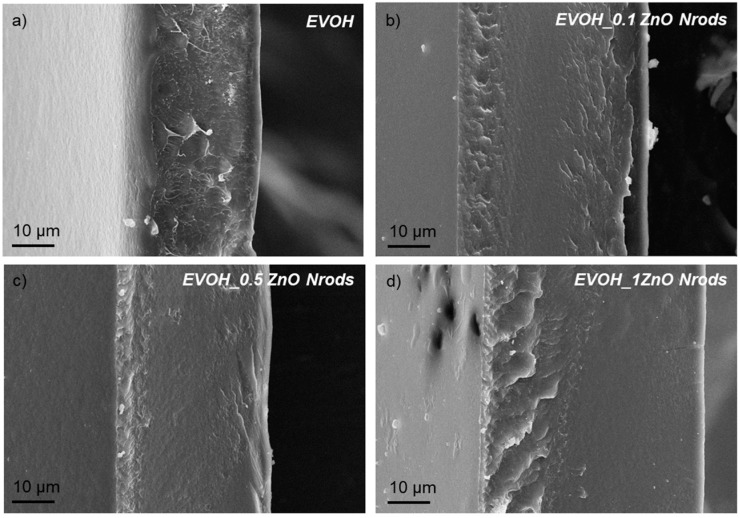
FESEM images of fractured surfaces for EVOH and EVOH_ZnO Nrods-based systems.

Figure 2 shows the morphological investigation of the fractured surfaces of EVOH-based films analyzed by FESEM. The FESEM analysis was carried out to evaluate the effect on microstructure of ZnO Nrods and the different concentration of nanofillers (0.1, 0.5, and 1 wt%) with respect to the polymeric matrix utilized to produce the different EVOH-based systems.

The fractured surface of EVOH-based film appeared smooth, homogeneous, and uniform (Figure 2a) [39]. The homogeneous surface highlights the good processability and film-forming characteristics of EVOH films. The addition of ZnO Nrods at different concentrations (Figure 2b–d) did not affect the neat EVOH cross-section structure (Figure 2a). The EVOH_ZnO Nrods-based systems showed smooth and homogeneous fractured surfaces; similar behavior was observed in literature combining ZnO nanoparticle in poly(lactic acid)-based films [40].

**Figure 3:**
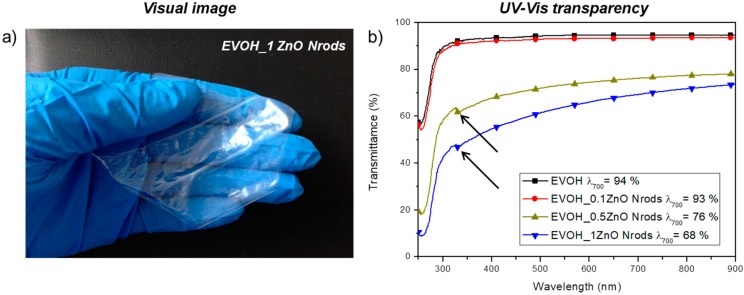
Visual images of poly(vinyl alcohol-co-ethylene) (EVOH)_1ZnO Nrods film (**a**) and UV-Vis analysis of EVOH-based formulations (**b**).

Figure 3a,b shows visual observation of EVOH_1ZnO Nrods film and UV-Vis transmission spectra of EVOH and EVOH nanocomposite thin films, respectively.

EVOH thin film (Figure 3b) is a transparent system, with a transmittance value of 94% at a visible wavelength of 700 nm. All the EVOH nanocomposites combined with zinc oxide Nrods maintain good levels of transparency from 450 to 900 nm (Figure 3b), a slight reduction in terms of transparency was registered for EVOH-based systems loaded with the high content of ZnO Nrods.

Specifically, the transmittance at a visible wavelength of 700 nm for EVOH_ZnO Nrods films were in the range of 93%–68% (transmittance (%): EVOH_0.1ZnO Nrods_λ=700nm_ = 93%, EVOH_0.5ZnO Nrods_λ=700nm_ = 76%, and EVOH_1ZnO Nrods_λ=700nm_ = 68%). The formulations containing ZnO Nrods absorb UV light starting at around 400 nm down to 250 nm with a drastic reduction of transparency (Figure 3b), the minimum transparency is found at 250 nm [37].

The transparency reduction in EVOH_0.5ZnO Nrods and EVOH_1ZnO Nrods at 326 nm (Figure 3b, see the arrows) is due to the light absorption of ZnO Nrods [41].

The color and gloss parameters of EVOH and EVOH_ZnO Nrods-based formulations are summarized in Table 1. The colorimetric and gloss investigations have been performed to evaluate the influence of zinc oxide nanorods before and after the accelerated-aging test (Time = 5 and 10 days).

EVOH and EVOH_ZnO Nrods-based formulations have been characterized by high and similar lightness value (L∗) and color parameters (a*and b* coordinates). All of the different samples are transparent and homogeneous in accordance to the transparency measurements (Figure 3a,b). This behavior highlights how the presence of ZnO Nrods does not affect the color of different formulations.

Regarding EVOH-based films formulations before and after accelerated-aging test (Time = 5 and 10 days), it is possible to underline that ΔE^∗^ value for EVOH films has been obtained after 10 days in accelerated aging conditions (ΔE∗ = 2.23 ± 0.25). For EVOH_ZnO Nrods-based formulations before and after the accelerated-aging test (Time = 5 and 10 days), similar color parameters (L,*a*, and b*) have been registered (Table 1). Concluding, the accelerated-aging test did not change significantly the color parameters of EVOH-based formulations.

This result could be considered related to the high optical characteristics and resistance of both polymeric matrix and zinc oxide nanofillers, that act as efficient absorber of UV radiation [42,43]. Improved stability of EVOH can be due to the UV blocking capability of ZnO nanoparticles, which diminish the amount of harmful UV rays reaching the polymeric system, leading to less deterioration of the polymer matrix. The gloss values of EVOH and EVOH_ZnO Nrods-based systems are summarized in Table 1. The presence of zinc oxide nanofillers in EVOH films before the accelerated-aging test did not induce variation in terms of gloss parameters (gloss at time 0 days: EVOH = (157 ± 2)°, EVOH_0.1ZnO Nrods = (154 ± 4)°, EVOH_0.5ZnO Nrods = (157 ± 1)°, and EVOH_1ZnO Nrods = (158 ± 3)°). The accelerated-aging test at different times (Time = 5 and 10 days) induces a reduction of gloss parameters vs time.

**Figure 4:**
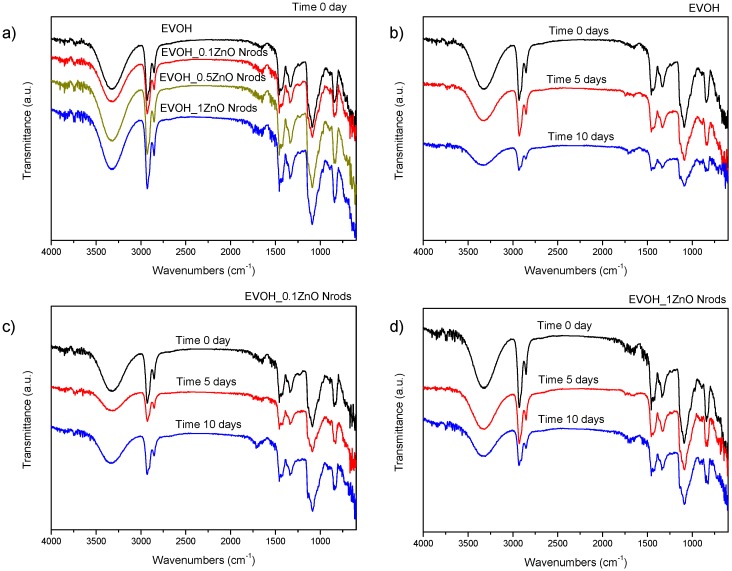
FT-IR spectra of EVOH and EVOH_ ZnO Nrods at different ZnO contents at time 0 (a), and after different exposition times (5 and 10 days) of accelerated-aging test for EVOH (**b**), EVOH_0.1ZnO Nrods (**c**) and EVOH_1ZnO Nrods (**d**).

In Figure 4, the FTIR spectra of neat EVOH and EVOH_ZnO Nrods sample, before and after UV weathering, are reported. Prominent and characteristic peaks can be seen in the FTIR spectrum of neat EVOH: in detail, it is possible to observe the presence of characteristic OH stretching (3329 cm^−1^), CO (1453 cm^−1^), and –CH_2_–CH_2_– stretching in the 2931 cm^−1^ asymmetric stretching and 2853 cm^−1^ symmetric stretching region, the peak at 1418 cm^−1^ due to –CH_2_ asymmetric stretching) and 1339 cm^−1^ (–CH asymmetric stretching), 1090 cm^−1^ (C–O stretching); 850 cm^−1^ (–CH out-of-plane deformation) [44].

The presence of the ZnO Nrods did not alter the main features of the EVOH spectrum: in particular, infrared spectra of the EVOH samples with different ZnO contents were analyzed to evaluate possible changes in molecular organization (Figure 4a). The prominent peak at 3329 cm^−1^ is due to (O–H) stretching of hydroxyl groups which shifts to 3320 cm^−1^, thus suggesting an interaction between the hydroxyl groups of polymer and ZnO nanoparticles [45]. Differences in the crystalline content were investigated by comparing the intensities for the bands at 1418 and 1090 cm^−1^. The band at 1418 cm^–1^ arises from all-trans conformation crystallizable chain segments, the vast majority of which are presumed to exist within a crystalline environment, whereas the broad envelope at 1090 cm^–1^ arises from the contribution of at least one amorphous band at 1113 cm^–1^ [15,33]. No significant differences were found in these bands among samples: this indicates that the amount of crystalline fraction in the polymer may not be altered even if high concentrations of ZnO Nrods are incorporated. After UV weathering (Figure 4b), FTIR measurements were performed to examine if any chemical changes occurred within the polymer: an apparent reduction in intensity and a weak shift of the broad band centered at 3329 cm^−1^ with increasing exposure time of the samples was observed [46]. This band was assigned to EVOH hydrogen bonded O–H stretching vibrations and its broadness is thought to arise from a distribution of hydrogen bonded O–H groups of varying strength and geometry, with a shift related to physical transformation and change of dipolar and other intermolecular interactions [47]. The overall variation indicated a general weakening of hydrogen-bonding with irradiation time [48].

The same trend was observed also for EVOH_0.1ZnO Nrods and EVOH_1ZnO Nrods (Figure 4c,d): while UV light is known to have a rather limited aging effect on EVOH polymers, in the case of ZnO-containing nanocomposites, some other changes that support transformation of OH groups are noticed in the spectra at about 1338 and 1454 cm^−1^, variations being associated with O–H deformation modes. Moreover, the vibration at around 1091 cm^−1^ related to C–O stretching also appears to decrease. Summarizing, these changes provide evidence that the ZnO presence in the OH-rich hydrophilic zones of the EVOH polymer, where the degradation starts, leadts to the formation of –CO moieties [49,50].

### 3.3. Thermal Characterizations of EVOH and EVOH_Nrods-Based Systems

**Figure 5:**
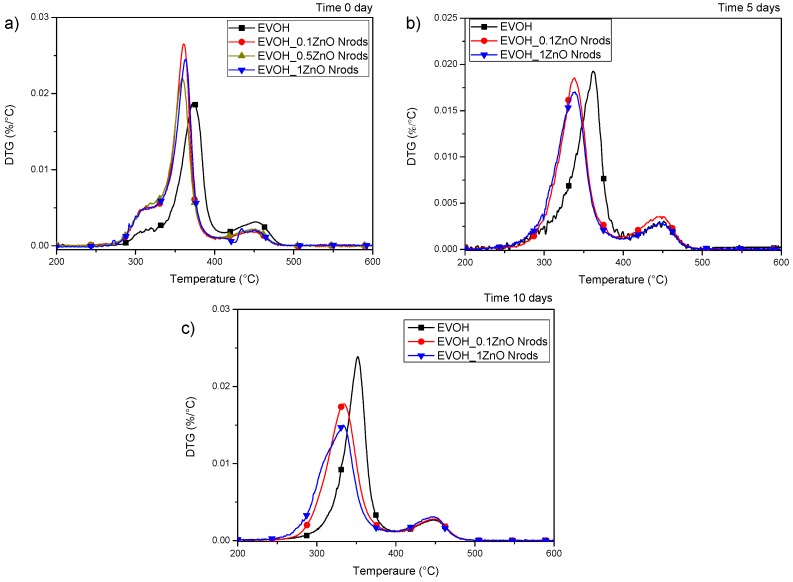
Derivative curves (DTG) of EVOH-based formulations before, and after different exposition times during the accelerated-aging test. (**a**) time = 0 (**b**) 5 days; (**c**) 10 days.

Figure 5a shows the derivative curves (DTG) of neat EVOH and ZnO Nrods-based nanocomposite films, characterized by the presence of a multi-step degradation behavior.

The Neat EVOH system shows two main degradation steps (and a shoulder of the main peak), the first one at 370–390 °C, attributed to the major component fraction, poly(vinyl alcohol), while the second one was at a higher temperature of around 450 °C, which can be attributed to the presence of ethylene [11,51]. Although all ZnO containing samples show similar thermal behavior, exhibiting two main stages of degradation, they showed a reduced thermal stability with respect to neat EVOH for the poly(vinyl alcohol) component, with a decrease of the main peak temperature from 374 to 361 °C: this is an indication that the oxide causes a reduction in the thermal stability of the polymer in some compositions [52]. The addition of ZnO nanoparticles to EVOH leads to a reduction in the thermal stability of the polymeric matrix, which is in direct correlation with the concentration of nanoparticles, therefore, as the ZnO content increases, the thermal stability for EVOH decreases. It is reported for polymer nanocomposites that the addition of ZnO to various polymer matrices can lead to either stabilizing or degradation effects [53], even in the case of PVA-based materials [54,55]: in the present case, ZnO Nrods acted as a catalytic agent in produced nanocomposites [56], with accelerated decomposition kinetics (peak of maximum degradation rate at 0.019% and 0.025%/°C, respectively for neat EVOH and EVOH_1ZnO Nrods), accompanied by the release of polymer–ZnO links, which may be responsible for the anticipated decomposition event.

The marked decrease in thermal stability upon ZnO loading arises from the fact that, as other metal such as Al, Sn and ZnO catalyzes EVOH depolymerization and this effect is much more marked in the case of nanocomposite containing rod-shaped NPs, as already observed by Lizundia et al. [57]. Due to the fact that the catalytic reactions take place at the ZnO interfaces, the amount of ZnO-EVOH interfaces would determine the thermal degradation behavior of nanocomposites.

In Figure 5b,c, the effect of UV weathering of thermal stability of the same systems (neat EVOH, EVOH_0.1ZnO Nrods and EVOH_1ZnO Nrods), respectively after 5 and 10 days, has been evaluated: it has been shown that aging of EVOH in the presence of a radiation source causes degradation through the formation of carbonyl, hydroperoxide, and hydroxyl groups, due to the preferential targeting of the H-C-OH sites by oxygen [44]; in addition it has been shown that weight-loss rate is faster at short times (0.017%/°C at 5 days for EVOH_1ZnO Nrods) and slows down for longer times (0.015%/°C at 10 days for EVOH_1ZnO Nrods). It is also clear from the DTG curves profiles that the ZnO nanoparticles essentially showed the same catalytic role even during the weathering treatment, strongly lowering the thermal stability of related nanocomposites (a decrease from 361 °C at time 0 up to 338 and 334 °C, respectively at 5 and 10 days, was measured for the EVOH_1ZnO Nrods system), even if a slight decrease with respect of neat EVOH was measured (a decrease from 374 °C at time 0 up to 362 and 352 °C, respectively at 5 and 10 days, was measured for the EVOH), confirming the delaying effect in the release of volatile low-molecular-weight photoproducts, due to nanoparticles accumulation [8,21].

**Figure 6:**
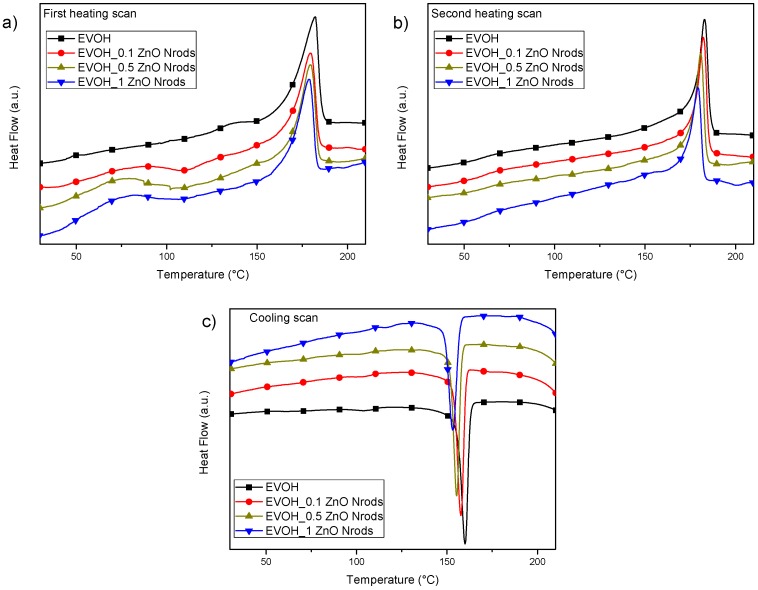
DSC thermograms of EVOH-based formulations at first heating scan (**a**), cooling scan (**b**) and second heating scan (**c**).

In order to evaluate the effect of ZnO Nrods presence and content on the crystallization and melting phenomena of copolymer matrix, thermal properties of EVOH-based formulations films have been also analyzed by DSC. In Figure 6a–c, the thermograms of neat EVOH sample and nanocomposites (1st heating, cooling, and 2nd heating scan) at the different content of ZnO Nrods are reported. During the first scan (Figure 6a), it was observed that glass transition temperature of EVOH_ZnO Nrods nanocomposites increases as the content of ZnO is increased up to 1% wt., confirming a restricted mobility of EVOH molecules in the presence of nanofillers, resulting from the strong hydrogen bonding between the oxygen-containing functional groups on the ZnO surface and hydroxyl groups of EVOH molecules [58].

On the other hand, no significant differences in the melting temperature of the nanocomposites in comparison with neat material were detected, while changes in melting enthalpy were measured, affecting the overall degree of crystallinity, with a decrease of the Xm value from 40.9% up to 30.7% for EVOH_1ZnO Nrods. This result can confirm the fact that ZnO nanofillers dispersed in the EVOH matrix confined the mobility of the EVOH chains close to the ZnO, as already observed with the Tg increase, hindering the regular packing of the EVOH chains into crystal lattices [59]. Data obtained from cooling scan (Figure 6c and Table 2) highlighted the presence of two crystallizations peaks, the first one at around 105 °C for neat EVOH film, and the second one centered at a high temperature (160 °C): in general, during the cooling scan, the crystallization temperature and enthalpies decreased, for both peaks, increasing the concentration of ZnO in the matrix. In such nanocomposites, the ZnO Nrods inhibited the nucleation and crystal growth of the EVOH: as a consequence of the cooling enthalpy decrease, even the crystallization degrees were reduced, having the lower value of Xc registered for the sample EVOH_1ZnO Nrods containing the higher amount of ZnO Nrods (Xc = (28.6 ± 0.6)%). The trend for glass transition was similar to the one detected for the first heating, with a visible effect in the enhancement for EVOH_1ZnO Nrods system. During the second heating scan, a general decrease of the Tm and the ΔHm values was registered in the presence of ZnO, confirming the behavior already observed in the first heating scan. The melting temperatures showed a decrease after addition of ZnO nanoparticles because of a decrease in lamellar thickness of the polymer crystals in comparison with neat EVOH: the changes in the melting behavior can be explained by the fact that, because of the good interaction between the ZnO nanoparticles and poly (vinyl alcohol-co-ethylene), the mobility of the polymer chains was restricted [60].

### 3.4. Mechanical Characterizations of EVOH and EVOH_Nrods Systems

**Figure 7:**
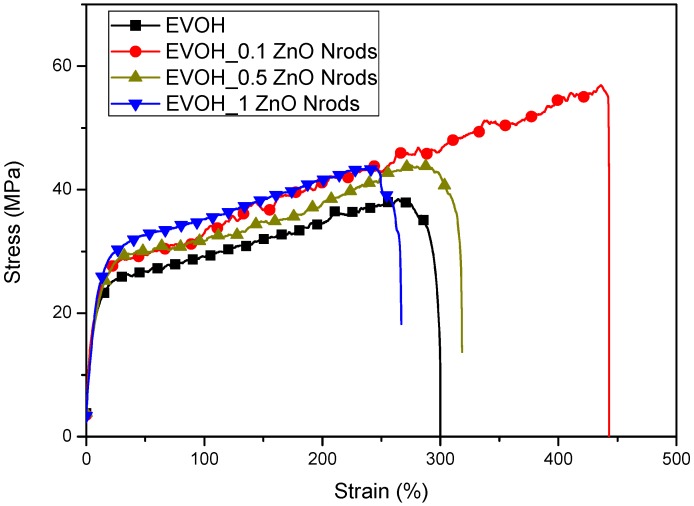
Stress–strain curves of EVOH-based formulations.

The presence of ZnO Nrods in EVOH matrix was analyzed also in terms of mechanical properties. Mechanical parameters of EVOH and EVOH_ZnO Nrods thin films were evaluated from stress–strain curves obtained performing tensile tests at RT. Data are summarized in Table 3, while the stress–strain curves are reported in Figure 7.

EVOH and EVOH_ZnO Nrods-based formulations show an evident ductile behavior (Table 3 and Figure 7). The presence of zinc oxide nanorods added at higher concentrations (0.5 wt% and 1 wt% ZnO Nrods) respect to EVOH content provoked a clear reduction of Young’s modulus (*E*_Young_(EVOH) = (440 ± 65) MPa, *E*_Young_(EVOH_0.1ZnO Nrods) = (415 ± 30) MPa, *E*_Young_(EVOH_0.5ZnO Nrods) = (345 ± 80) MPa, and *E*_Young_(EVOH_1ZnO Nrods) = (300 ± 10) MPa) a similar phenomenon was already observed in literature for polymeric systems containing ZnO nanoparticles as a reinforcement [26,40,61]. The presence of 1% wt of ZnO Nrods in EVOH film determined a reduction of mechanical performance in terms not only of Young’s modulus (*E*_Young_), but also in terms of deformation at break (ε_b_) and strength at break (σ_b_) with respect to EVOH-based film, highlighting the brittle nature induced in the EVOH matrix by the presence of a high content of ZnO nanorods. EVOH_0.1ZnO Nrods represented the ideal formulation in terms of mechanical performance required in packaging applications, showing modulated properties in elastic and plastic mechanical response (Table 3 and Figure 7). Specifically, EVOH_0.1ZnO Nrods showed higher strength and deformation at break and similar values of Young’s modulus with respect to EVOH film (Table 3). The mechanical performance for EVOH-based film loaded with 0.1 ZnO Nrods can be related also to the smooth, homogeneous, and uniform fractured surface observed by FESEM and to the reduced crystallinity values detected by thermal investigation (Figure 2).

It may be possible to introduce higher amounts of nanoparticles into the composite to further reduce UV erosion during accelerated weathering conditions, but additional increment of loading could cause detrimental effect due to the agglomeration of nanosized particles.

### 3.5. Moisture Content and Oxygen Transmission Rate of EVOH_Nrods

Determination of the moisture content that the different polymeric systems can absorb in specific environmental conditions, and the oxygen transmission rate are two important issues in the packaging sector. The evaluation of oxygen transmission rate permits estimation of the oxygen transfer from the internal or external environment through the polymeric package wall, influencing the main characteristics resulting in a continuous change in the product quality of packaged products [62,63]. The specific barrier requirements depend on the product characteristics and the intended end-use application [64]. Table 4 shows the moisture content (MC) of EVOH and EVOH_ZnO Nrods-based formulations after 1 and 5 storage weeks at 53% RH and 25 °C. The test permits estimation of the MC that the different produced films could adsorb in specific conditions of temperature and humidity. The MC of the EVOH film was (1.51 ± 0.08)% and (1.55 ± 0.15)% after 1 or 5 weeks of storage, respectively. The results underlined that the equilibrium was reached in the first week of storage according to the literature [11].

The presence of zinc oxide nanorods in EVOH-based formulations induces a slight reduction of MC. This phenomenon is related to the homogeneous ZnO dispersion and also to the hydrophobic behavior of selected nanofillers [64]. After 5 weeks of storage, the MC data for all different produced formulations was kept unchanged with respect to the results registered after 1 week of storage (Table 4): this effect could be due to the saturation phenomena; similar data are registered in literature by using poly(vinyl alcohol) and EVOH-based films combined with active ingredients [11,32,65,66].

Table 5 summarizes the oxygen transmission rate of EVOH and EVOH_ZnO Nrods-based films.

The presence of zinc oxide nanorods induces an increase of the oxygen transmission rate with increasing amount of nanofillers in the polymeric matrix, mainly due to the increased amorphous portion of the nanocomposites films, that makes the polymer more permeable, as already revealed by the delayed crystallization step and reduced crystallinity values for the ZnO Nrods-containing films. On the other hand, other authors observed that the presence of zinc oxide nanoparticles, having a different aspect ratio, could induce even a strong reduction of oxygen permeability values in different polymeric-based nano-formulations [64,67], confirming how variant morphologies of the spherical or rod-like nanoparticles clearly induces a different diffusion pathway for the gas molecules.

## 4. Conclusions

Poly(vinyl alcohol-co-ethylene) (EVOH) films containing zinc oxide nanorods (ZnO Nrods) at 0.1, 0.5, and 1 wt%, have been realized by solvent casting. Results from tensile tests showed increased values for strength and deformation at break in EVOH-based formulations reinforced with 0.1 and 0.5 wt% of zinc oxide nanorods, indicating a system with 0.5% wt. ZnO Nrods as a promising composition, while results from colorimetric and transparency investigation underlined how the presence of ZnO Nrods in the EVOH copolymer did not induce evident alterations. Results from thermal and spectroscopic characterization confirmed the restricted mobility and hindered regular packing of the EVOH chains into crystal lattices in the presence of nanofillers, with a detectable increase of glass transition temperatures and crystallinity decrease for EVOH_ZnO Nrods nanocomposites with increasing ZnO Nrods content. In addition, the colorimetric test after the accelerated-aging test confirmed the possibility of using of these materials in the flexible packaging sector, especially in the fields where a high barrier to UV and oxygen is required.

## Figures and Tables

**Table 1:** Color coordinates of EVOH-based systems before and after different exposition times during the accelerated-aging test.

Formulations	L*	a*	b*	∆E*	Gloss (°)
**White Control**	99.47 ± 0.00	−0.08 ± 0.01	−0.08 ± 0.01	-	121 ± 0
**Time 0 day**					
**EVOH**	98.78 ± 0.06	−0.09 ± 0.01	0.14 ± 0.00	0.72 ± 0.06	157 ± 2
**EVOH_0.1ZnO Nrods**	98.21 ± 0.03	−0.10 ± 0.01	0.14 ± 0.00	1.28 ± 0.03	154 ± 4
**EVOH_0.5ZnO Nrods**	98.32 ± 0.13	−0.08 ± 0.00	0.20 ± 0.02	1.19 ± 0.14	157 ± 1
**EVOH_1ZnO Nrods**	98.78 ± 0.02	−0.10 ± 0.00	0.22 ± 0.02	0.79 ± 0.02	158 ± 3
**Time 5 days**					
**EVOH**	98.12 ± 0.20	−0.09 ± 0.01	0.28 ± 0.05	1.40 ± 0.18	138 ± 4
**EVOH_0.1ZnO Nrods**	97.40 ± 0.25	−0.06 ± 0.02	0.18 ± 0.05	2.09 ± 0.25	136 ± 5
**EVOH_1ZnO Nrods**	97.28 ± 0.11	−0.11 ± 0.01	0.45 ± 0.06	2.25 ± 0.09	132 ± 2
**Time 10 days**					
**EVOH**	98.18 ± 0.02	−0.22 ± 0.01	0.86 ± 0.10	2.23 ± 0.25	137 ± 4
**EVOH_0.1ZnO Nrods**	97.47 ± 0.33	−0.22 ± 0.01	0.86 ± 0.10	2.04 ± 0.10	137 ± 4
**EVOH_1ZnO Nrods**	97.71 ± 0.02	−0.21 ± 0.02	0.87 ± 0.01	2.01 ± 0.03	147 ± 2

**Table 2:** Differential scanning calorimetry (DSC) data of EVOH base systems.

**Formulations**	**First Heating Scan**					
	***T*_g_ (°C)**	**ΔH_m_ (J g^−1^)**	***T*_m_ (°C)**			***X*_m_ (%)**
**EVOH**	48.8±0.1	82.8 ± 0.4	180.7 ± 2.2			40.9 ± 0.2
**EVOH_0.1ZnONrods**	59.5 ± 0.1	67.6 ± 2.4	179.8 ± 0.7			33.4 ± 1.2
**EVOH_0.5ZnONrods**	59.6 ± 0.1	66.5 ± 0.1	179.5 ± 0.3			33.0 ± 0.1
**EVOH_1ZnONrods**	57.4 ± 0.3	61.5 ± 2.7	178.0 ± 1.0			30.7 ± 1.3
	**Second Heating Scan**					
	***T*_g_ (°C)**	Δ***H***_**m**_ **(J g**^**−1**^**)**	***T***_**m**_ **(°C)**			***X***_**m**_ **(%)**
**EVOH**	64.6 ± 0.3	83.2 ± 0.1	182.9 ± 0.1			41.1 ± 0.1
**EVOH_0.1ZnONrods**	63.5 ± 0.1	70.3 ± 1.5	181.9 ± 0.3			34.8 ± 0.7
**EVOH_0.5ZnONrods**	66.0 ± 2.3	68.2 ± 0.3	178.7 ± 2.7			33.8 ± 0.1
**EVOH_1ZnONrods**	64.7 ± 0.1	64.7 ± 0.1	178.7 ± 0.7			30.8 ± 0.1
	**Cooling Scan**					
	***T*_g_ (°C)**	Δ***H***_**c**_ **(J g**^**−1**^**)**	***T***_**c**_ **(°C)**	Δ***H***_**c**_ **(J g**^**−1**^**)**	***T***_**c**_ **(°C)**	***X***_**c**_ **(%)**
**EVOH**	61.0 ± 0.7	2.2 ± 0.2	104.9 ± 0.4	71.8 ± 0.4	159.9 ± 0.2	36.6 ± 0.3
**EVOH_0.1ZnONrods**	60.4 ± 0.7	1.2 ± 0.1	104.1 ± 0.8	60.1 ± 2.5	157.7 ± 0.1	30.3 ± 1.2
**EVOH_0.5ZnONrods**	60.6 ± 0.7	1.2 ± 0.1	102.0 ± 0.4	62.8 ± 0.4	155.2 ± 0.3	31.8 ± 0.3
**EVOH_1ZnONrods**	65.2 ± 0.7	0.9 ± 0.1	101.6 ± 0.1	56.3 ± 1.1	152.5 ± 0.9	28.6 ± 0.6

**Table 3:** Mechanical properties of EVOH-based systems.

Formulation	Mechanical Properties		
	**σ_b_ (MPa)**	**ε_b_ (%)**	**E_Young_ (MPa)**
**EVOH**	45 ± 4	265 ± 28	440 ± 65
**EVOH_0.1ZnO Nrods**	55 ± 3	410 ± 30	415 ± 30
**EVOH_0.5ZnO Nrods**	50 ± 9	365 ± 100	345 ± 80
**EVOH_1ZnO Nrods**	40 ± 6	240 ± 15	300 ± 10

**Table 4:** Moisture content of EVOH-based systems.

Formulations	MC (%) @ 1 Week	MC (%) @ 5 Week
**EVOH**	1.51 ± 0.08	1.55 ± 0.15
**EVOH_0.1ZnO Nrods**	1.50 ± 0.06	1.60 ± 0.15
**EVOH_0.5ZnO Nrods**	1.45 ± 0.02	1.55 ± 0.10
**EVOH_1ZnO Nrods**	1.35 ± 0.0.5	1.43 ± 0.07

**Table 5:** Oxygen transmission rate.

Formulations	OTR (cm^3^ m^−2^ days^−1^)	e (mm)
**EVOH**	1.97	0.08
**EVOH_0.1ZnO Nrods**	2.74	0.06
**EVOH_0.5ZnO Nrods**	4.79	0.06
**EVOH_1ZnO Nrods**	6.10	0.06
